# Aspects of Gut Microbiota and Immune System Interactions in Infectious Diseases, Immunopathology, and Cancer

**DOI:** 10.3389/fimmu.2018.01830

**Published:** 2018-08-15

**Authors:** Veronica Lazar, Lia-Mara Ditu, Gratiela Gradisteanu Pircalabioru, Irina Gheorghe, Carmen Curutiu, Alina Maria Holban, Ariana Picu, Laura Petcu, Mariana Carmen Chifiriuc

**Affiliations:** ^1^Department of Microbiology and Immunology, Faculty of Biology, University of Bucharest, Bucharest, Romania; ^2^Earth, Environmental and Life Sciences Section, Research Institute of the University of Bucharest, Bucharest, Romania; ^3^National Institute for Diabetes, Nutrition and Metabolic Diseases Prof. Dr. N. Paulescu, Bucharest, Romania

**Keywords:** gut microbiota, opportunistic infections, autoimmunity, chronic inflammation, cancer, antibiotics, probiotics, diet

## Abstract

The microbiota consists of a dynamic multispecies community of bacteria, fungi, archaea, and protozoans, bringing to the host organism a dowry of cells and genes more numerous than its own. Among the different non-sterile cavities, the human gut harbors the most complex microbiota, with a strong impact on host homeostasis and immunostasis, being thus essential for maintaining the health condition. In this review, we outline the roles of gut microbiota in immunity, starting with the background information supporting the further presentation of the implications of gut microbiota dysbiosis in host susceptibility to infections, hypersensitivity reactions, autoimmunity, chronic inflammation, and cancer. The role of diet and antibiotics in the occurrence of dysbiosis and its pathological consequences, as well as the potential of probiotics to restore eubiosis is also discussed.

## Introduction

The microbiota consists of a multispecies microbial community living within a particular niche in a mutual synergy with the host organism. Besides bacteria, the microbiota includes fungi, archaea, and protozoans (1, 2), to which viruses are added, which seem to be even more numerous than microbial cells (3). The gastrointestinal tract (GIT), with its epithelial barrier with a total area of 400 m^2^, is a complex, open, and integrated ecosystem with the highest exposure to the external environment. The GIT contains at least 10^14^ microorganisms belonging to >2,000 species and 12 different phyla, the associated microbiome containing 150- to 500-fold more genes than the human DNA (1, 4–7). The GIT microbiota exhibits a huge diversity, being individually shaped by numerous and incompletely elucidated factors, such as host genetics, gender, age, immune system, antropometric parameters, health/disease condition, geographic and socio-economical factors (urban or rural, sanitary conditions), treatments, diet, etc. (8, 9). Recent metagenomic data demonstrated that the majority of component species is not present in the same time and in the same person, but, however, few species are abundant in healthy individuals, while other species are less represented (4, 7). In addition to the distribution along the digestive tract segments, the GIT microbiota of the three distinct transversal microhabitats, i.e., floating cells in the intestinal lumen, cells adherent to the mucus layer and respectively to the surface of the epithelial cells, is also different (3).

Recent findings suggest that the microbial colonization of the GIT starts before birth, as revealed by the placental microbiome profile, being composed of members of Firmicutes, Proteobacteria, Tenericutes, Bacteroidetes, and Fusobacteria groups, which were found to share some similarities with the human oral microbiome (10). Also, the meconium of full term infants is not sterile, harboring 30 genera normally found in the amniotic fluid, vagina, and the oral cavity (8, 9, 11). We can assume that the bacteria reach these sites mainly from the vaginal tract, although selective translocation is also possible. Archaea were also detected in the vaginal microbiota of pregnant women, accounting for a mother-to-child transmission (12).

Vaginally born infants have a microbiota containing species derived from the vaginal microbiota of their mothers. Conversely, in the case of cesarean section delivered babies, the microbiota is similar to the skin microbiota and is rich in *Propionibacterium* spp. and *Staphylococcus* spp. (13).

It is generally accepted that the pregnancy period and the first 1,000 days after birth are the most critical timeframes for interventions and any modulation made at this point has the potential to improve child growth and development (14). Delivery mode seems to influence immunological maturation through microbiota development. Cesarean section delivered children were found to have a higher number of antibody-secreting cells (11).

Furthermore, the human milk is involved in the GIT microbiota and immune system development. In addition to its nutritional components, this natural functional food contains numerous bioactive substances and immunological components that control the maturation of the newborn intestine and the composition of the microbial community. Numerous studies revealed that breast-feeding has a protective role in infants, conferred by a complex mixture of molecules, including lysozyme, sIgA, alpha-lactalbumin, lactoferrin, but also free oligosaccharides, complex lipids, and other glycoconjugates (14). The proteolytic processing of glycoprotein k-casein, with the release of glycomacropeptides, prevents colonization of the gut by pathogens, through competition with the receptors of the gut epithelial cells in breast-fed infants. Lactoferricin is a potent antimicrobial agent, explaining the decreased infant death rate caused by gastrointestinal and respiratory infections in breast-fed infants (14, 15). Moreover, breast milk contains ~10^9^ bacterial cells/L (16) and prebiotic oligosaccharides (fructans) which stimulate the multiplication of *Bifidobacterium* spp. and *Lactobacillus* spp., while follow-on milk powder stimulates proliferation of enterococci and enterobacteria (17, 18). As the infant grows, solid foods are introduced, therefore the microbiota diversity increases, and the microbiota community evolves toward the adult-like state. Although some dominant enterotypes represented by *Bacteroides, Prevotella*, and *Ruminococcus* genera are recognized, however, the final composition of the adult microbiota is unique and the factors guiding this feature are still a matter of debate (19).

The very active microbial community has been shown to mutually interact with the host and to exert a lot of beneficial roles, explaining its tolerance by the host organism. The GIT microbiota is involved in energy harvest and storage, and, due to its particular metabolic pathways and enzymes, it extends the potential of the host metabolism. This property is believed to exhibit a potent evolutionary pressure toward the establishment of bacteria as human symbionts (11). The GIT microbiota influences the normal gut development, due to its ability to influence epithelial cell proliferation and apoptosis of host cells. Although the intimate interactions between microbiota and host cells are widely unknown, a major mechanism seems to involve short-chain fatty acids (SCFA), resulted from the fermentation of indigestible polysaccharides (fibers), such as butyrate, acetate, and propionate with an important anti-inflammatory role. SCFAs also support intestinal homeostasis in the normal colon, by aiding intestinal repair through the promotion of cellular proliferation and differentiation. However, SCFAs seem to inhibit the cancerous cells proliferation. Among the different SCFAs, butyrate has a paramount role in intestinal homeostasis due to its role as a primary energy source for colonocytes (20, 21). In addition, the GIT microbiota stimulates the nonspecific and specific immune system components development, just after birth and during the entire life and it acts as an antiinfectious barrier by inhibiting the pathogens’ adherence and subsequent cellular substratum colonization and by the production of bacteriocins and of other toxic metabolites. Moreover, the microbiota is predominantly composed of anaerobes which prevent the process of translocation of aerobic/facultatively anaerobic bacteria and the consecutive systemic infections in immunodeficient individuals. Importantly, some GIT microbiota representatives (*Escherichia coli* and *Bacteroides fragilis*) are involved in the synthesis of vitamins, such as B1, B2, B5, B6, B12, K, folic acid, and biotin. Also, the GIT microbiota has the ability to degrade xenobiotics, sterols and to perform biliary acids deconjugation (*B. fragilis* and *Fusobacterium* spp.) (19).

All these aforementioned effects are occurring when the microbiota community is characterized by an interspecies balance, known as eubiosis. Any perturbation of eubiosis, known as dysbiosis, could become a pivotal driver for various infectious and non-infectious diseases, each of them with specific microbiota signatures that can further trigger pathophysiologies in different organs (11).

Our aim was to review these physiological roles, focusing on one side the GIT microbiota contribution to the immune system development and education, and on the other side, to what is happening when eubiosis is replaced by the dysbiosis status; in this case the immunostasy is altered, the host becomes more susceptible to infections, both exogenous and endogenous; immunotolerance is affected and the immune system will react against the self-components (autoimmunity), or vary in intensity, being either over (allergic reactions and chronic inflammation) or less/inappropriately (immunodeficiency and cancer) activated.

## GIT Microbiota and Immune System Development

The mucosal immune system is highly specialized, its functions are largely independent of the systemic immune system (15) and it undergoes major changes after bacterial colonization of the intestinal tract (22).

Commensal microorganisms are required for the maturation of the immune system, which “learns” to differentiate between commensal bacteria (which are becoming almost quasi-self and tolerated antigens) and pathogenic bacteria (23, 24). Toll-like receptors (TLRs) from the membrane of the epithelial and lymphoid cells of the small intestine are involved in this differential recognition, being responsible for the normal development of the intestinal mucosal immune system. TLRs suppress the occurrence of an inflammatory response and promote immunological tolerance to normal microbiota components. The role of TLRs is to recognize different general microbe-associated molecular patterns (MAMPs) [containing various bacterial antigens (e.g., peptidoglycan components—muramic acid, capsular polysaccharides and lipopolysaccharides, flagellin and unmethylated bacterial DNA CpG motifs)] and to trigger the innate intestinal immunity (25, 26). Following stimulation, a complex cascade of signals is initiated, leading to the release of nuclear factor kappa-light-chain-enhancer of activated B cells (NF-kB), which activates a variety of genes coding for chemokines, cytokines, acute phase proteins, and other effectors of the humoral immune response (27, 28). TLR activity decreases during the first weeks of life, potentially allowing the development of a stable gut bacterial community. Furthermore, TLR activation by antigens belonging to the normal intestinal microbiota is signaling the inhibition of inflammatory reactions, being thus essential to maintain intestinal homeostasis (29). Complementarily, NOD-like receptors (NLRs) recognize various microbial specific molecules and trigger the assembly of inflammasomes, which can act as sensors of damage-associated patterns. The NLPRP6 deficiency has been associated with an altered immune response (e.g., decreased IL-18 levels), dysbiosis, and intestinal hyperplasia (11, 30).

Gastrointestinal tract microbiota has been shown to modulate neutrophil migration and function (31) and to affect the differentiation of T cell populations into different types of helper cells (Th), respectively: Th1, Th2, and Th17 or into regulatory T cells (Tregs) (25). The Th17 cells are a subset of TCD4^+^ cells, which secrete multiple cytokines (IL-22, IL-17A, and IL-17F), with a significant impact on immune homeostasis and inflammation (32, 33). Unlike Th1 and Th2 cells, which have a stable secretory profile after differentiation, Th17 cells retain divergent cytokine expression profiles and functions (34). It has been shown that the administration of the purified capsular polysaccharide from the commensal bacterium *B. fragilis* suppresses the production of IL-17 and protects the colonic mucosa against inflammatory reactions initiated by bacterial antigens, stimulating TCD4^+^ lymphocytes to produce IL-10 (35). On the other side, the colonic environment also stimulates *de novo* differentiation and expansion of peripherally derived regulatory T cells from naïve CD4^+^ T cells (36). Tregs are key mediators of immune tolerance, limiting an inappropriately high inflammatory response (37), their dysfunction leading to autoimmune disorders (38).

sIgA has a crucial role in the local immune response, being considered the first line of defense against pathogens and toxins. sIgA production specific to different mucosal antigens is following their capture by Peyer’s patches M cells, transformation by underlying antigen-presenting cells [dendritic cells (DCs)], activation of T cells, and ultimately B cell class switch recombination in mesenteric lymph nodes (MLNs) and gut-associated lymphoid tissue. The commensal antigens induce the production of low amounts of sIgA through the modulation of their immunodominant epitopes, thus harboring an advantage for the colonization of the intestinal niche (11). A set of cytokines, including TGF-β, IL-4, IL-10, IL-5, and IL-6 stimulates IgA production. Some of these cytokines, notably IL-10 and TGF-β are crucial in maintaining the mucosal tolerance, therefore proving the link among sIgA production, immunity, and intestinal homeostasis (39).

In individuals with dysbiosis, immune responsiveness could be upregulated to promote the development of a more optimal status. This could be obtained through specific effects of sIgA, or less specific effects of innate immunity effectors (such as defensins) or local environment changes (i.e., diarrhea). In case of diarrhea, the host eliminates undesirable microbial communities in order to prepare niches for recolonization with more beneficial microbial populations, as a last resort to healing (14).

The host-commensal microbiota communication triggers antimicrobial responses from the epithelium including the release of several antibacterial lectins, including RegIIIc, α-defensins, and angiogenins (40, 41). These antibacterial effectors reduce the amount of potentially pathogenic microbes and provide protection against subsequent abnormal immune responses. For instance, *Bacteroides thetaiotaomicron* triggers the production of antimicrobial peptides which target other intestinal microbes. The microbiota of mice expressing a human enteric α-defensin, DEFA5, has no segmented filamentous bacteria (42), which are responsible for inducing IL-17-producing Th17 cells, which have been correlated with inflammatory bowel disease (IBD) and colorectal cancer.

Furthermore, aberrant microbial development during maturation of the innate immune system leads to defective immunological tolerance, which subsequently promotes exacerbated autoimmune and inflammatory diseases (e.g., allergen-induced airway hyperreactivity) (3). Microbial products may induce chronic stimulation of immune responses, leading to chronic, non-resolving inflammation and tissue damage, particularly after mucosal injury.

## GIT Microbiota and Infections of Exogenous or Endogenous Origin

### Microbiota–Pathogen Interactions

As also named “the last undiscovered human organ,” the intestinal microbiome has an impact on immune system development and differentiation. In addition, the microbiome holds a paramount role in the initiation and progression of infectious diseases (43).

Through the colonization of the mucosal entry sites of pathogens, microbiota could directly prevent the invasion by foreign microbes—a process known as colonization resistance (by competing with pathogenic bacteria in the gut for adhesion sites and nutrients, but also by releasing toxic molecules to counteract pathogen colonization), as well as indirectly, through the stimulation of the immune response. As stated above, the gut microbiota provides signals to stimulate the normal development of the immune system as well as the maturation of immune cells (44–46). The microbiota stimulates the secretory IgA response that is involved in inactivating rotaviruses, competes *Clostridium difficile* colonization, and neutralizes cholera toxin (47). Moreover, the signaling molecules released by the microbiota actively shape the host systemic immune response by regulating haematopoesis, and consequently potentiating the response to infection (48). Signals derived from the commensal microbiota trigger the development of granulocyte/monocyte progenitors in the bone marrow and hence affects tissue-resident innate immune populations which in turn promotes the early host innate response. In line with this, the absence of the microbiota derived signaling molecules cause alterations in tissue-resident myeloid populations prior to infection and leads to susceptibility to systemic infection by *Staphylococcus aureus* and *Listeria monocytogenes* (49).

The synergic interactions of the innate immune system and microbiota could be also exploited by pathogens to evade the antiinfectious mucosal barrier. A suggestive example is given by the oral bacterium *Porphyromonas gingivalis*, which escapes the host immune response *via* TLR2 signaling pathway modulation leading to dysbiosis and subsequent inflammation (50). Also, some viruses are able to interfere with the interplay between bacteria and the innate immune system (i.e., TLR4 signaling), for guaranteeing their efficient transmission (51). It has been proved that the antiviral host response is improved by antibiotic depletion of commensal microbiota. Intestinal antiviral innate immunity is the result of the induction of IL-18, interferon (IFN)-λ, or IL-22 pathways, which promote the expression of signal transducer and activator of transcription 1 (STAT1) and antiviral genes. Although IL-22 and IL-18 are both stimulated by commensal bacteria, IFN-λ expression is inhibited by the microbiota, hence enabling viral persistence. It has been also clearly demonstrated that interactions between gut epithelial cells and microbiota are crucial to maintain barrier defenses and gut homeostasis. For instance, the microbiota has a role in maintaining tight junctions’ integrity which limits *Salmonella typhimurium* invasion (52). On its turn, the intestinal pathogen *S. typhimurium* induces IL-22 production which targets commensal bacteria and liberates a colonization niche for the pathogen.

Generally, the antiinfectious barrier is efficient when the microbiota is complex and stable, in a eubiotic status. On the contrary, when dysbiosis occurs (due to different causes, e.g., poor colonization, antibiotherapy or an unbalanced, unhealthy diet, different pathological conditions leading to secondary immunodeficiencies), the microbiota loses its antiinfectious barrier potency and the host can be easily infected with different pathogenic microorganisms from the environment. In addition, some species of microbiota, enriched in the new condition of dysbiosis, can manifest their pathogenic potential by producing opportunistic infections. For example, antibiotics can be used for treating certain pathological GIT diseases, but the induced alteration of the intestinal microbiota could lead to metabolic disturbances, such as increased intestinal permeability, and may also increase susceptibility to infections [e.g., fungal and *Clostridium difficile* infections (CDIs)].

Recent findings proved a clear correlation between microbiome composition and risk of infectious diseases. For example, microbiota composition represents an infection risk for *Plasmodium falciparum* infection, and also a key factor for diverse vaccine responses (43).

Recent studies aiming to investigate the specific role of gut microbiota and immune system interactions in infectious diseases focused mainly on microbiome manipulation. This was achieved either by probiotics administration or fecal microbiota transplantation. Serious conditions which are prevalent in children, such as necrotizing and acute infectious diarrhea, but also antibiotic-associated diarrhea, CDIs and ventilator-associated pneumonia could be treated more efficiently by microbiota manipulation, with a better outcome, reduced mortality, and faster recovery rates. Since microbiota manipulation could control the balance between health and infectious disease, intestinal microbiota alteration by a pathogen or a pathobiont can lead to chronic diseases. *In vivo* studies demonstrated that the colonization of adherent-invasive *Escherichia coli* (AIEC, an *E. coli* pathovar involved in Crohn’s disease pathogenesis) during microbiota acquisition drove chronic colitis in mice (53). It seems that AIEC, *Yersinia enterocolitica* and probably other pathobionts, may promote chronic inflammation in susceptible hosts by producing gut microbiota alterations which lead to a higher capacity in activating innate immunity/pro-inflammatory gene expression (54). A recent study by Inoue et al. shed light on the impact of hepatitis C virus (HCV) infection on the gut microbiota. Unlike healthy individuals, HCV infected patients showed dysbiosis characterized by a decrease in Clostridiales and enrichment in *Streptococcus* and *Lactobacillus* genera. Microbiota alterations were present even in patients with mild liver disease, as revealed by the transient increase in Bacteroides and Enterobacteriaceae (55).

Gastrointestinal tract microbiota members can translocate from the digestive mucosa and reach the general circulation, indirectly by stimulating IL-12 production by splenic macrophages, DCs, which, in turn, regulates the Th1/Th2 balance toward a cell-mediated Th1 response (56). Studies have shown that the soluble products of *Lactobacillus fermentum* DSMZ 20052 determine the decrease of IL-8 levels by inhibiting the NF-kB pathway, thus alleviating the pro-inflammatory effect induced by *Yersinia enterocolitica* infection (57). Other studies support the activation of NF-kB signaling pathway with the subsequent activation of inflammatory genes by some probiotics. One of the hallmarks of NF-kB activation is the production of IL-6 (56). It has been shown that the colonization of the digestive tract of germ-free rats with *Bifidobacterium lactis* BB 12 strain stimulates the IL-6 synthesis (58).

### Microbe–Microbe Interactions (Quorum Sensing)

All bacteria are able to communicate with each other by signaling molecules, which allow the bacterial cells to sense the environment, monitor population density and to adjust accordingly their gene expression. Through this type of communication, bacteria acquire an advantage crucial for dissemination and survival in highly competitive environments, which harbor hundreds of coexisting species (e.g., the oral cavity, the intestine). Depending on the involved members, intercellular communication is divided into two categories, based on the quorum-sensing (QS) mechanism. QS is a density-dependent molecular language responsible for the regulation of cellular phenotype/behavior as a response to environmental changes. The first type is the intraspecific cell-to-cell communication though specific QS molecules and the second mechanism consists of the interspecific communication based on an universal chemical “language,” which provides interspecific signaling between bacteria and eukaryotic/host cells. QS is orchestrated by small molecules, usually considered hormone-like organic molecules called autoinducers (AIs). AIs are represented by diffusible molecules called homoserine-lactones (Acyl-HSL) in Gram-negative bacteria and not diffusible peptidic molecules (AIP) in Gram-positive ones. A universal interspecies signal (“cross talk”) which contains AIs common for both Gram-positive and Gram-negative bacteria has been identified in 55 pathogens so far. These compounds depend on the microbial cellular density and hold a paramount role in various niches, especially in highly colonized sites, such as the gut and the oral cavity (59). This mechanism of communication regulates the expression of virulence genes in pathogens, with an important role in infection. For example, a relatively low virulence factors production by a limited population of bacteria may promote a robust host response that neutralizes these molecules, while the coordinated virulence factors gene expression by high-density bacterial populations can lead to higher secretion of extracellular factors (60, 61). The produced molecules have also an immunomodulatory effect, controlling the inflammatory response which can induce severe damaging of host tissues (62). Recent studies reported they may have also a therapeutic potential, for autoimmune diseases as immunosuppressive drugs (63). The QS mechanism allows bacteria to regulate the host colonization by commensal bacteria and to modulate the host response (64–66). Although the specific mechanism(s) through which AIs influence mammalian cells is unclear, a modified immune response was observed. For example, the 3-oxododecanoyl homoserine lactone (HSL-C12) induces apoptosis and Ca^2+^ release from endoplasmic reticulum stores. HSL-C12 has also been reported to modulate the inflammatory signaling (67), being immunosuppressive at or below 10 µM concentrations, but pro-inflammatory and proapoptotic at 20 µM and above (68). HSL-C12 acts through TLR- and Nod/Ipaf/caterpillar-independent signaling and activates multiple NF-κB-associated pro-inflammatory genes including IL-1α, IL-6, IL-8, Cox2, mPGES, PGE2, and MUC5AC in different cell types. The pro-inflammatory effects may be achieved through activation of MAPKs, extracellular-signal-regulated kinases, inhibition of peroxisome proliferator-activated receptor γ, or Ca^2+^ (69). In the presence of pro-inflammatory molecules, such as lipopolysaccharides (LPS) or TNFα, HSL-C12 may inhibit NF-κB signaling and expression of pro-inflammatory cytokines in macrophages and epithelial cells (69). *In vivo* experiments proved that direct injection of HSL-C12 in C57BL/6 mice lead to the expression of macrophage inflammatory protein-2 (MIP-2) (the mouse analog of the human cytokine IL-8) and also other cytokines. Significantly, higher concentration of MIP-2 was found in mice infected with QS active microbial strains than those inoculated with the QS-deficient bacteria (70).

Quorum-sensing is also used by microbiota members in order to detect the presence of other similar microbes (71); their well-known antiinfectious barrier effect is to the result of the antagonistic relationships with pathogens; is well known that probiotic strains are able to produce antimicrobial molecules as well as small QSIs which are interfering with the QS mechanism and virulence expression of the pathogens (72–74). It seems that the antimicrobial eosinophil-derived neurotoxin, cathelicidins, defensins, AI2 signaling molecules hold paramount functions in intra- and interspecies communication.

Certain intestinal mammalian hormones mimic the action of bacterial signaling molecules, thus increasing the complexity level of the bidirectional communication between bacteria and the host (75). In this context, a particular field of the exchange of molecular information between the many microorganisms and the host (76) is represented by microbial endocrinology, defined by the ability of GIT microbiota to orchestrate a bidirectional communication with the central nervous system by producing and sensing neurochemicals that are derived either within the microorganisms themselves or within their host (77). Steroid hormones (adrenaline and noradrenaline), due to their ability to pass through the plasmatic membrane are involved in the inter-kingdom communication between microorganisms and their mammalian host (78, 79). Although bacteria do not express adrenergic receptors, some studies indicate that bacterial cells are responsive to adrenaline and/or noradrenaline (NA) and recent studies suggest they have an important impact in maintaining the homeostasis of gut microbiota (80). The existing data sustain that NA may work as a siderophore (81). It is believed that NA is involved in overexpression of enterobactin and in the iron chelating mechanism in *E. coli*, subsequently increasing the bacterial growth rate. On the other hand, gut microbiota can produce neurochemicals with hormonal activities that could extend beyond the gut, being involved in the modulation of anxiety, depression, cognition, pain, inflammatory, autoimmune, and metabolic diseases (82–87).

## Role of GIT Microbiota in Autoimmune and Inflammatory Diseases

The alteration of the complexity and eubiotic state of microbiota might promote intestinal and extraintestinal autoimmune and inflammatory disorders (type I diabetes, rheumatoid arthritis, ankylopsing spondilosis, IBD, pulmonary disease, atopy, non-alcoholic fatty liver disease, obesity, atherosclerosis, carcinogenesis, etc.) although the mechanisms involved are not well understood (3). Many researchers reported an opposite connection between the incidence of immune disorders and the infectious process. Within this line of thought, children under the age of 5 years living in developed countries are not exposed to many of the microbes, compounds and antigens they would have encountered a century ago. This lack of early immune stimulation by biotic factors that humans and their ancestors have evolved with may hinder the functioning of the immune system later in life and lead to hypersensitivity, autoimmune, or inflammatory diseases (88).

### Type 1 Diabetes

Initially called juvenile-onset diabetes, type 1 DM (T1DM) is a chronic illness associated with high morbidity and premature mortality. This disease is caused by the patient’s inability to secrete insulin as a result of the autoimmune destruction of the pancreatic beta cells (89). Usually, T1DM occurs early in life, but recent studies reported that up to 50% of new-onset T1DM patients are older than 20 years (90). The major factor in the pathophysiology of T1DM is represented by autoimmunity. The genetically susceptible individuals (around 95% of patients with T1DM) harbor either human leukocyte antigen DR3-DQ2 or DR4-DQ8 haplotypes, or have the UBASH3A mutation, also known as STS2, located on chromosome 21, which are linked also with other autoimmune diseases, such as celiac disease (91, 92), viral infections (mumps, enterovirus, coxsackie virus B4, and rubella), but also toxic chemicals, exposure to cytotoxins or cow’s milk in infancy and may stimulate the production of antibodies against antigenically similar beta cell molecules. T1DM is associated with a low diversity of microbiota and with the expansion of distinct groups of bacteria (93, 94). However, human studies have not yet elucidated the causal relationship between the gut microbiome and pathogenesis of T1DM. Some models have linked the gut microbiome with the development of T1DM, respectively, the Hygiene Hypothesis, the Leaky Gut Hypothesis, the Perfect Storm Hypothesis, and the Old Friends Hypothesis. Based on the Leaky Gut Hypothesis, the increased permeability of the intestinal epithelium develops from loss of tight barrier function (95). Macromolecules derived from diet and microbial antigens are able to pass through the epithelial barrier and consequently trigger intestinal inflammation that could lead to pancreatic beta cell attack (95). The Old Friends Hypothesis sustains the role of commensal microbes which have evolved together with their host and highlights that loss of these commensal microbes may impact the host’s immune response regulation and homeostasis (96). On the other hand, the Perfect Storm Hypothesis reunites aspects from the Leaky Gut Hypothesis and the Old Friends Hypothesis advocating that a combination of both increased intestinal permeability an altered microbiota composition, and an impaired intestinal immune responsiveness interact together culminating in anti-islet autoimmunity (97). The Hygiene Hypothesis was formulated by David Strachan (1989) who, trying to explain the actual high incidence of allergic and autoimmune diseases, postulated that increasing T1DM incidences is the result of a diminished or a lack of contact with infectious agents due to elevated hygienic conditions (98). In a recent study by Maffeis et al. on children at risk of developing T1DM, increased intestinal permeability was correlated with microbiota alterations. Unlike healthy controls, children with T1DM risk exhibited high levels of *Globicatella sanguinis, Dialister invisus*, and *Bifidobacterium longum* (99). In addition, it was also reported that the *Bacteroidaceae* family is enriched in children with T1DM. Moreover, T1DM children exhibited a decrease of *Bifidobacterium pseudocatenulatum* and *Bifidobacterium adolescentis* (100). A subsequent study revealed that the microbiota of genetically predisposed infants from 3 months to 3 years old was characterized by an enrichment of *Rikenellaceae, Ruminococcus, Streptococcus*, and *Blautia* as well as by reduced alpha diversity (101).

### Rheumatoid Arthritis

Recent studies found a correlation between rheumatoid arthritis, the enrichment of *Prevotella copri* and colitis susceptibility, suggesting that the inflammatory component of autoimmune diseases might be modulated by an impaired communication between the host and the microbiota (102). These data are also sustained by a recent study which characterized the gut microbiota of DBA1 mice after collagen induction arthritis (CIA) and found altered distribution of the microbiota. Mice susceptible to CIA harbored *Lactobacillus* as the dominant genus prior to the onset of arthritis. During disease progression, the operational taxonomic units (OTUs) of the *Lachnospiraceae, Bacteroidaceae*, and S24-7 families were significantly elevated in CIA-susceptible mice. Also, germ-free mice receiving microbiota harvested from CIA-susceptible mice presented an elevated induction of arthritis compared to those receiving microbiota from CIA-resistant mice (103).

### Celiac Disease

Modification of the normal gut microbiota may have a role in the onset and/or progression of celiac disease. Species such as *Staphylococcus epidermidis, Staphylococcus pasteuri*, and *Klebsiella oxytoca* were enriched in duodenal biopsies harvested from patients diagnosed with active celiac disease. Species such as *Streptococcus mutans* and *Streptococcus anginosus* were reduced in patients with celiac disease compared to healthy people, independently of the inflammatory status. Fucosyltransferase 2 (FUT2) gene regulates the expression of ABH blood group antigens in mucus as well as other body secretions and also influences the composition of mucosa-associated bacteria. A mutation in FUT2 gene lead to decreased bacterial heterogeneity and abundance, including a lower quantity of *Bifidobacterium* spp., in the human gut. Fut2-deficient mice presented more susceptibility to *Candida albicans* colonization comparing to wild-type mice and *Candida albicans* infection is a culprit in the onset of celiac disease. *Bifidobacterium* spp. were shown to have a protective role against *C. albicans* colonization. Therefore, alteration of the microbiota due to mutation of FUT2 gene decreases colonization resistance and has a role in the pathogenesis of celiac disease (104).

### Inflammatory Bowel Disease

Aberrant immune responses against commensal bacteria may promote the development of IBDs, such as ulcerative colitis and Crohn’s disease, providing experimental models for studying different aspects of the immune system-microbiota crosstalk, such as oxidative stress, microbial sensing, and antigen processing. Some alleles of the genes encoding for innate immunity mechanisms, i.e., ATG16L1, which is involved in autophagy; NOD2, which is connected to the activation of the immune system by peptidoglycans; and CLEC7A, linked with the recognition of fungi by DCs have been shown to predispose to IBD (105). Dysbiosis controls the pathogenesis of IBD, affecting over one million people in the United States and one-quarter million in the UK (106). The IBD pathogenesis involves the bacterial adherence to the gut mucosa and invasion into mucosal epithelial cells leading to the occurrence of an inflammatory response, mediated by the production of TNF-α by monocytes/macrophages. This chronic bowel inflammation affects the epithelial cell tolerance to intestinal bacteria leading to changes in intestinal microbiota composition with an increase in aerobic bacteria accompanied by a significant decrease in the fecal levels of butyric and propionic acid in IBD patients. However, despite the generally accepted involvement of LPS in triggering an inflammatory effect (2), the main species adhering to the mucosa surrounding the colon mucus layer are *Bifidobacterium* spp. and *Clostridium coccoides*, suggesting that IBD is not triggered by a microbial species, but by an unbalanced microbiota. The hydrogen peroxide-producing colonic bacteria have been also suggested as causative agents of IBD in young adults (107). The studies performed on a mouse model of colitis (dextran sodium sulfate-induced colitis) showed that the introduction of anaerobic, noncultivatable segmented filamentous bacteria stimulates Th17 development, while commensals such as *Bacteroides fragilis* or *Clostridium* species, facilitate the differentiation of regulatory T-cell and IL-10 production in the gut. In most cases of spontaneous colitis models, including the IL-10^−/−^ mouse, antibiotics or a germ-free state have been shown to prevent the development of colitis (24). The presence of Gram-positive bacteria, such as Lachnospirillaceae seems to be necessary for the infiltration of colitogenic macropahages and monocytes into the colon through induction of C–C chemokine receptor type-2 ligands, as revealed by the decrease of inflammatory reaction in mice treated with vancomycin (24). In humans, *Faecalibacterium prausnitzii* is one of the most abundant colonic bacteria found within the fecal mass but is also present in the adjacent mucosa, representing 5–20% of the total fecal microbiota of healthy individuals. Low counts of *Faecalibacterium prausnitzii* have been linked to several pathological disorders including Crohn’s disease (108).

### Allergic Diseases

Allergic diseases affect more than half a billion people worldwide. The development of allergy is clearly associated with some genetic and molecular factors but environmental factors including the gut microbiota are also involved. Indeed, reduced microbial diversity in infancy was correlated with an increased risk for allergies later in life. The commensal microbiota was reported to provide protection against allergic airway inflammation and food allergy (109) since mice treated with antibiotics and germ-free mice developed an exacerbated disease. TLR2- or TLR4-deficient mice develop pulmonary damage after chronic intake of a high-fat diet, a feature that can be transmitted to wild-type mice by fecal transplantation (110).

Recent data proved that lower prevalence of bacteria such as *Akkermansia, Faecalibacterium*, and *Bifidobacterium*, along with higher abundance of fungi such as *Rhodotorula* and *Candida* in neonates may lead to allergy susceptibility by modulating T-cell differentiation (48).

### Systemic Lupus Erythematosus (SLE)

Systemic lupus erythematosus is a systemic autoimmune disease with unknown etiology characterized by the presence of hyperactive and aberrant antibody response to nuclear and cytoplasmic antigens (111). The dysbiosys observed in SLE is characterized by an increase of the *Bacteroides* phyla and a decrease in the *Firmicutes* (112). Despite the fact that the role of microbiota in the development of SLE is poorly understood, it is suggested that the dysbiosis observed in the SLE patients could be related to this disease. In line with this, mouse models of lupus exhibited an accelerated development of the disease that was linked to increased levels of *Lachnospiraceae* and low levels of *Lactobacillaceae* (113).

### Skin-Related Autoimmune Pathologies

Skin autoimmune diseases have also been linked to microbiome shifts. For instance, a recent study by Scher et al. compared the composition of gut microbiota in patients with psoriatic arthritis or with psoriasis to that of healthy controls (114). The gut microbiota signature of psoriatic arthritis and psoriasis groups exhibited decreased bacterial diversity and a reduced relative abundance of *Ruminococcus, Pseudobutyrivibrio*, and *Akkermansia*. Importantly, the microbiota profile of psoriatic arthritis was similar to that of IBD patients, therefore suggesting a link between the gut microbiota and this skin disease (114). In case of scleroderma, most patients suffer from GIT symptoms that may be due to changes in intestinal microbiota composition. Recently, Volkmann et al. (115) revealed that *Firmicutes* were highly abundant in systemic sclerosis patients compared to healthy controls, whereas *Bacteroidetes* were lower in one of the cohorts, compared to healthy controls (115).

### Neurological Inflammatory Diseases

In the last decade, more studies proved that the gut microbiota has an impact on brain development and function. The studies compared germ-free and conventional laboratory rodents and the results suggest that the absence of microbiota alters anxiety-like behavior and also enhances the hypothalamic pituitary adrenal system stress reactivity. This abnormal behavior observed in germ-free animals was eradicated if the intestinal microbiota was restored in early life but not in the adulthood stage, suggesting the existence of a critical period of time for microbiota imprinting on stress responsiveness. The mechanism of action is not completely understood. Neuroactive bacterial metabolites are transported through the bloodstream to the brain and stimulate entero-endocrine cells or the vagus nerve, or modulate the immune system and, subsequently the inflammatory status, proving that dysbiosis could impact anxiety-related disorders of the treatment of anxiety-prone rodents with antibiotics or probiotics exhibited an anxiolytic-like activity (116). For example, there are studies involving the gut microbiota in host cognition or *Alzheimer’s disease* (*AD*)-related pathogenesis. Species from gut microbiota can generate large amounts of amyloids and LPS, which may modulate various signaling pathways and the synthesis of pro-inflammatory cytokines involved in AD pathogenesis. Furthermore, imbalances in the gut microbiota can lead to inflammation that is associated with AD (117). Microbial dysbiosis was also seen in the gut of multiple sclerosis (MS) patients and significant differences in microbiota composition between patients with MS and healthy controls were observed. Patients with active disease exhibited reduced species richness, whereas the microbiota of patients in remission was similar to that of the healthy controls. Comparing treated and untreated MS patients, certain genera such as *Sutterella* (*Proteobacteria*) and *Prevotella* (*Bacteroidetes*) were found to be reduced in untreated patients but restored after treatment. However, in treated patients *Sarcina* spp. was also reduced, a fact that proves the potential of MS therapies to alter the gut microbiome. As in case of MS, neuromyelitis optica (NMO) is driven by pathogenic Th17 cells reactive against self-proteins, in this case aquaporin-4 (AQP4) which is a channel protein transporting water across cell membranes. AQP4 is expressed by brain astrocytes and shows sequence homology with an ATP-binding cassette transporter permease from *Clostridium perfringens*. AQP4-specific T cells cross-react with *Clostridium perfringens*. Significant compositional differences were observed between the microbiome of patients with NMO compared to healthy controls: *Clostridium perfringens* and *Fibrobacteres* were enriched in NMO patients and together may have an impact on disease progression (118).

## Gut Microbiota and Immune System Interactions in Cancer

Chronic inflammation could drive carcinogenesis (e.g., colorectal cancer in patients with untreated IBD, hepatocellular carcinomas following chronic hepatitis). Through epithelial injury and inflammation, chronic infections (viruses, *Helicobacter pylori* and other *Helicobacter* spp., *Bacteroides fragilis, Bacteroides vulgatus, Escherichia coli, Citrobacter rodentium, Citrobacter freundii*, and protozoa) are linked to carcinogenesis with approximately 18% of the worldwide cancer burden (119). However, cancer, as well as other diseases, is not attributable to a single pathogen but to overall microbiome changes. Dysbiosis of gut microbiota leads to an increase in bacterial populations that stimulate tumorigenesis and the loss of protective ones. Inflammation might augment community-level alterations in the microbiota and aid the bacterial translocation into the neoplastic tissue, which in turn promotes the expression of inflammatory cytokines subsequently leading to tumor growth. The colonic microbiota may also promote colorectal cancer by stimulating exaggerated immune responses (i.e., *via* Th17 cells) (120). The dysbiosis caused by a deficiency of the NLRP6 inflammasome promotes cancer development *via* IL-6-induced epithelial proliferation (121, 122).

### Microbiota In Oral Cancers

Oral cancer, particularly oral squamous cell carcinoma (OSCC) which evolves from the lining mucosae of the lips and the mouth is a multifactorial disease caused environmental factors (tobacco, human papillomavirus, and alcohol consumption) and host genetics (123). The microbiota shifts linked to OSCC have been analyzed in several studies. The culture-based analysis of surface swabs revealed that the levels of *Fusobacterium* spp. and *Porphyromonas* spp. were significantly higher in the OSCC tissue compared with the adjacent healthy mucosa (124). Subsequently, it was reported that sections of gingival squamous cell carcinoma harbored higher levels of *Porphyromonas ginigivalis* (125). A study targeting the differences in bacterial counts in 45 OSCC samples and 229 healthy controls revealed that cancer samples exhibited elevated *Capnocytophaga gingivalis, Streptococcus mitis*, and *Prevotella melaninogenica* (126). Another bacteria, *Streptococcus anginosus* has been linked to all head and neck squamous cell carcinoma, including OSCC (127). Subsequent studies by Sasaki et al. and Morita et al. detected *Streptococcus anginosus* in 45 and 13% OSCC samples, respectively (128, 129). Importantly, recent studies have revealed that *Streptococcus anginosus* is found in non-tumorous oral tissue even in higher levels compared with tumoral tissue, thus implying that *Streptococcus anginosus* is a normal inhabitant of the oral microbiota (130). Hooper et al. used culture methods as well as 16S rRNA Sanger sequencing to isolate 108 bacterial species from within the tissue of OSCC biopsies including *Ralstonia insidiosa, Fusobacterium naviforme, Peptostreptococcus micros, Clavibacter michiganensis subspp. tessellarius, Fusobacterium naviforme, Micrococcus luteus, Prevotella melaninogenica, Staplylococcus aureus, Exiguobacterium oxidotolerans*, and *Veillonella parvula* (131). These studies revealed that bacteria have tumor specificity, since several species were found in either the non-cancerous or cancerous sites. Pushalkar et al. analyzed 10 specimens of OSCC by 16S rRNA Sanger sequencing and detected 35 novel species. Nevertheless, this study identified in the tumors a totally different panel of bacteria including *Eubacterium infirmum, Eubacterium brachy, Gemella haemolysans, Streptococcus gordonii, Peptostreptococcus stomatis, Gemella morbillorum, Streptococcus parasanguinis, Johnsonella ignava, Streptococcus salivarius*, and *Gemella sanguinis* (130). These differences were likely caused by the fact that conventional culture methods and Sanger sequencing have low depth of analysis; thus, these techniques cannot guarantee robust identification of possibly relevant low abundant species. The advent of next-generation sequencing (NGS) has overcome this caveat. Several studies have used NGS to investigate the OSCC-associated microbiome. Pushalkar et al. analyzed salivary samples from three OSCC cases and two healthy controls and identified 860 OTUs among which 244 were exclusively present in the OSCC. Thus, the genera *Gemella, Peptostreptococcus, Porphyromonas, Micromonas, Streptococcus, Rothia*, and *Lactobacillus* had a higher abundance in the OSCC samples, while *Capnocytophaga, Leptotrichia, Actinobacillus, Oribacterium Prevotella*, and *Neisseria* were prevalent in samples without cancer (132). Schmidt et al. investigated swabs of lesion surface and normal mucosa from 8 pre-cancer cases, 18 OSCC samples, and 9 cancer free controls and observed that the Bacteroidetes phylum was significantly enriched in cancerous and normal tissues of OSCC patients compared to pre-cancer and healthy controls. This suggests that elevated colonization with bacteria from this phylum may be considered a possible biomarker for OSCC risk. In addition, tumor samples were associated with significantly higher levels of *Fusobacterium* and reduced abundance of *Rothia* and *Streptococcus* (133). A subsequent study by Al-hebshi et al. employed additional bioinformatics analysis and showed that OSCC samples contained 228 bacterial species, among which *B. fragilis* (134). Recently, Guerrero-Preston et al. compared the saliva microbiota in DNA isolated from Oropharyngeal (OPSCC), Oral OCSCC patients, and normal epithelium controls. The authors characterized the HNSCC saliva microbiota before and after surgical resection and revealed a predominance of Bacteroidetes, Proteobacteria, and Firmicutes with low abundance of Actinobacteria and Fusobacteria before surgery. The most enriched genera were *Veillonella, Haemophilus, Streptococcus, Lactobacillus*, and *Prevotella* with lower levels of Neisseraceae *and Citrobacter*. HNSCC patients exhibited a significant loss in microbiota diversity and richness (135). A novel study by Zhao et al. revealed that a group of periodontitis-correlated taxa, including *Dialister, Fusobacterium, Peptococcus, Filifactor, Catonella, Parvimonas*, and *Peptostreptococcus* was more abundant in OSCC samples (136).

### Gastric Microbiota and Gastric Malignancies

Gastric cancer is one of the most common malignancies worldwide, gastrointestinal malignancies being responsible for about one-third of global cancer (137). The pathogenesis of this disease is a multi-stage process of affected by multiple factors still not clearly elucidated. Environmental factors, *Helicobacter pylori* infection and genetic factors are all involved in gastric cancer pathogenesis. *Helicobacter pylori* are closely linked to gastric cancer and whether other intragastric microbes participate in gastric cancer development requires further investigation (138). Emerging evidence suggests that other microorganisms exhibit a role in the pathophysiology of gastric cancer. This so called non-*Helicobacter pylori* bacteria that are enriched in a hypoacidic environment could potentially trigger carcinogenesis *via* different mechanisms, such as producing toxic metabolites, promoting inflammation, modifying stem cell dynamics, and stimulating cell proliferation (139). A recent study of the stomach microbiota in patients with gastric cancer revealed similarities with the stomach microbiota of patients with dyspepsia and a normal gastric mucosa (140). An analysis performed on two human populations with high and low gastric cancer risk in Columbia revealed two significantly more abundant OTUs, *Veillonella* spp. and *Leptotrichia wadei* in the high-risk area and 16 OTUs, including *Staphylococcus spp*. were more frequent in the low-incidence region (139). In addition, Ferreira et al. showed that gastric carcinoma is associated with a significant decrease in *Helicobacter*, and an increase in *Lactobacillus, Citrobacter, Achromobacter, Clostridium*, and *Rhodococcus* genera abundance. Interestingly, *Phyllobacterium*, a bacteria commonly found in plant roots, was enriched in gastric carcinoma samples (141).

### Colorectal Carcinogenesis (CRC)

**Figure 1:**
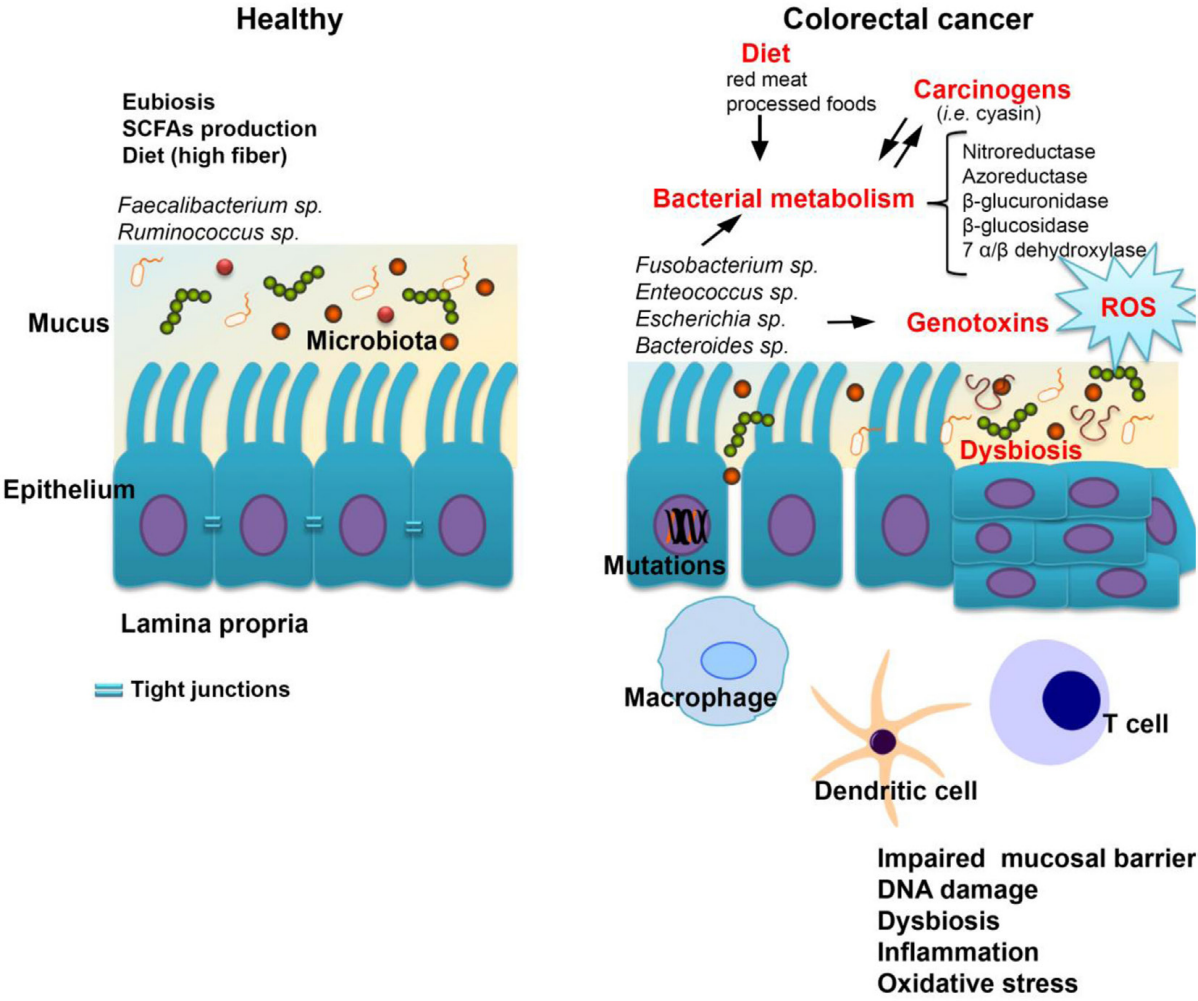
The host-microbiome interplay in colorectal cancer. Some bacterial species including *Fusobacterium* spp*., Enterococcus* spp., *Escherichia coli*, and *Bacteroides* spp. are most commonly associated with colorectal cancer. Changes in microbiota composition (dysbiosis) impair the gut barrier function of epithelial tight junctions and the mucus layer. Consequently, this increases the exposure of the epithelium to bacteria and their metabolites which may have carcinogenic potential. Bacterial translocation also leads to increased inflammation associated with the production of procarcinogens or toxic chemicals such as reactive oxygen species (ROS), bacterial genotoxins (colibactin), and hydrogen sulfide (H_2_S). Altogether, these changes trigger oxidative stress which leads to DNA damage. A series of pathways independent of the inflammatory response, but related to bacterial enzymes which could generate carcinogenic compounds, are cited in the literature by which the gastrointestinal microbiome may be involved in the genesis and evolution of neoplastic processes.

Colorectal carcinogenesis is caused by a combination of host- and microbiota-dependent mechanisms. In this equation, the most common environmental factors are lifestyle choices and diet. Unhealthy diets high in fat, red meat, alcohol, and low in fiber are associated with an increased risk of adenomas and CRC (142). In addition, smoking, gender, ethnicity, and lifestyle, obesity all impact CRC development (143, 144). Certain bacteria promote carcinogenesis directly *via* the secretion of substances that lead to DNA damage. Several outstanding examples include the release of reactive oxygen species by *Enterococcus faecalis*, the excessive production of nitric oxide from immune cells triggered by *Helicobacter hepaticus*, as well as enterotoxin secretion by enterotoxigenic *Bacteroides fragilis* (ETBF), which can activate the *c-MYC* oncogene. Also, the ETBF fragilysin toxin activates signaling pathways such as Wnt/β-catenin and NF-κB to induce excessive cell proliferation and inflammation (145). In addition, BFT could potentially generate a multi-step pro-tumorigenic signaling requiring NF-κB, IL-17R, and STAT3 in colonic epithelial cells leading to myeloid-cell-dependent distal colon tumorigenesis (146). Other species including *Parvimonas micra, Solobacterium moorei*, and *Peptostreptococcus anaerobius* were also significantly correlated with CRC. In a recent study by Tsoi et al., *P. anaerobius* was significantly elevated in biopsies from tumor lesions as well as in stool samples from CRC patients compared to healthy controls (147). The accumulation of hydrogen sulfide generated by sulfate reducing bacteria in response to a diet rich in meat, promotes chronic inflammation and the release of mutagenic compounds or genotoxins (such as CDTs produced by *Salmonella enterica* serovar *typhi* and *Escherichia coli* and colibactin, a secondary metabolite, a hybrid polyketide/nonribosomal peptide) (148, 149) (Figure 1).

In experimental models of CRC precancerous lesions were characterized by elevated levels of *Allobaculum* spp. and *Ruminococcus obeum*. These data suggest that modifications in the composition of adherent microbial populations may exhibit a role in adenoma development (150), by inducing a pro-inflammatory response (e.g., *Fusobacterium nucleatum* through its virulence factor FadA) (151). Novel studies identified additional virulence factors in *Fusobacterium nucleatum* (Fap2, LPS) that may act as triggers in the evolution from healthy epithelial cells to tumor cells (152).

It seems that microbial status may have an effect on tumorigenesis without direct influence on the inflammatory status. In line with this, up to 80% of IBD patients with long-standing disease (<30 years) do not develop colitis-associated CRC, hence highlighting that inflammation is not enough to trigger cancer (153).

It has been suggested that a higher microbial density in a sterile organ is correlated with a higher incidence of cancer, due to exposure to microbe-associated molecular patterns (MAMPs) and bacterial metabolites, while a decreasing microbiota in mice by antibiotics reduced the liver and colon cancer frequency (23).

However, microbiota can also exert anti-tumor effects in patients with sarcomas, by converting the tumor tolerance in an anti-tumor immune response, involving TLR agonists and NLR (154).

Microbiota can synthesize large amounts of folic acid, which plays a crucial role in regulating DNA synthesis, and other antioxidant substances such as selenium, vitamin C, and vitamin A, which help prevent DNA lesions. Some anaerobic species (*Clostridium orbiscindens* and *Eubacterium ramulus)* are involved in the process of colon and breast cancerogenesis, by degradation of some food glicozides and flavonoids with protective, antineoplasic effect (155). Methylazoxymethanol, a bioactive carcinogenic compound, the downstream active metabolite of plant glycoside cyasin is generated under the action of the bacterial β-glucosidase enzyme (156). This could explain the anti-tumoral effect of probiotic lactic acid bacteria including *Lactobacillus acidophilus* and *Lactobacillus casei* which express a decreased activity of azoreductase, β-glucuronidase, and nitroreductase (157). Butyrate producing bacteria also have anti-tumor effects in colon cancer cells by promoting cancerous cells apoptosis (2, 158). Butyrate induced apoptosis in colon cancer cell lines *in vitro* is correlated with its role as a histone deacetylase inhibitor, achieved through the modulation of different molecular pathways (159–162).

Since the gut microbiota is an important modulator of host immunity, it is natural to think that it could have an impact on the response to cancer therapy. As initially revealed by animal studies and further confirmed by human studies, the gut microbiome is an important factor in mediating the host response and toxicity to various anticancer therapies (e.g., immunotherapy with CpG oligonucleotides or chemotherapy). Commensal *Bifidobacterium* spp. enhances the tumor control in a similar way to programmed cell death protein 1 ligand 1-specific antibody therapy, through the augmentation of dendritic-cell function (163).

Patients with hematologic malignancies undergoing hematopoietic stem cell transplant (HSCT) are often treated with broad-spectrum antibiotics, immunosuppressants, and even total body irradiation thus dysbiosis is fairly common in these individuals (164, 165). After HSCT, dysbiosis is characterized by a loss of bacterial diversity and stability, reduced levels of *Faecalibacterium* and *Ruminococcus* and an enrichment of *Enterococcus, Streptococcus*, and Proteobacteria (166).

An improved overall survival after HSCT treatment was correlated with certain microbiota signatures characterized by higher levels of the genus *Blautia* (167). In addition, a higher density of *Eubacterium limosum* was linked to reduced relapse risk (168).

Conventional chemotherapy is also interacting with the immune system and the microbiota. For instance, during treatment with cyclophosphamide, the translocation of *Enterococcus hirae* and *Lactobacillus johnsonii* into MLNs can facilitate Th17 and Th1 responses. In addition, the effects of cyclophosphamide as well as other chemotherapy drugs were absent in antibiotic-treated or germ-free mice (169, 170). The impact of the microbiota was analyzed in case of treatment with immune checkpoint inhibitors targeting the immunomodulatory molecules found on the surface of T cells. Recent studies try to find means to control therapeutic resistance by identifying the predictors of the host response to immune checkpoint blockade (171, 172). The microbiota may impact the response to immune checkpoint inhibitors by targeting the programmed death receptor 1 (PD-1) and the cytotoxic T lymphocyte antigen 4 (CTLA-4) (163, 173).

Gopalakrishnan et al. reported that patients responsive to anti-PD-1 therapy harbored a higher microbial diversity characterized by higher Ruminococcaceae, *Faecalibacterium*, and Clostridiales relative abundance (174, 175). By contrast, nonresponders exhibited significantly lower bacterial diversity and a higher levels of Bacteroidales. In addition, the comparison between the composition of the gut microbiota with the immune profile in the tumor microniche showed that patients hosting a favorable gut microbiota exhibited enhanced antigen processing and presentation and elevated expression of cytolytic T cell markers compared to patients with unfavorable dysbiotic microbiota (166).

Toxicity scores were reported to be improved in anti-CTLA-4-treated mice after oral gavage with *Bacteroides fragilis* and *Burckholderia cepacia* (173). The impact of the microbiota on toxicity was also investigated in human cohorts (176–178). Anti-CTLA-4-treated melanoma patients without colitis showed enhanced levels of Bacteroidetes as opposed to those who did develop colitis (177). Additional studies revealed that patients with low abundance of Bacteroidetes and elevated *Faecalibacterium prausnitzii* and other related Firmicutes harbored an elevated risk of colitis in anti-CTLA-4 therapy (176, 178). Taxa within the Bacteroidales order (Bacteroidetes phylum) were linked to lack of responsiveness to immune checkpoint blockade, whereas their elevated abundance was correlated with a lower toxicity incidence (166, 174, 176–178). Nevertheless, at lower taxonomic levels several taxa within Firmicutes such as *Roseburia* and *Streptococcus* were linked with a lack of response (175, 178) whereas some taxa within Bacteroidetes (*Porphyromonas* and *Alistipes*) were correlated with response (164, 174, 175). In addition, other taxa were correlated with response (such as *Bifidobacterium longum, Collinsella aerofaciens, Akkermansia muciniphila*, and *Bifidobacterium adolescentis*) and non-response (such as *Gardnerella vaginalis* and *Actinomyces viscosus*) (164, 166, 174, 175, 178).

These studies might open up an appealing route of investigation for cancer prevention as well as to develop cancer therapeutics through microbiome manipulation.

## External Perturbations of Eubiosis Status

Time series data reveal that microbiota composition is relatively stable in case of healthy adult individuals over time. Two of the most important factors triggering changes in the microbiota are the dietary intake and the overuse of antimicrobials (179).

A recent study by Rothschild et al. investigated the interplay between environmental factors, host genetics and gut microbiota, and revealed that the gut microbiota community structure was mostly influenced by environmental factors rather than single nucleotide polymorphisms or genetic ancestry. In addition, only 1.9% of the gut microbiome is estimated to be heritable, whereas more than 20% of the microbiome β-diversity is shaped by the environment (diet, lifestyle, etc.) (180).

It was demonstrated that shifting to a high-sugar high-fat, “Western” diet from a plant polysaccharide-rich, low-fat or from a low-fiber/high-fat diet to a high-fiber/low-fat diet can change the mouse microbiota within a day (181) the monitoring of temporal dynamics of microbial communities within an individual through time could predict the disease states and help develop strategies to correct dysbiosis. Diet is also correlated with the gut enterotype, as proven by the fact that individuals eating a diet high in animal fat exhibit a *Bacteroides*-enterotype, whereas a carbohydrate-rich diet leads to a *Prevotella-*dominated enterotype (182). Several studies revealed that a diet rich in non-digestible carbohydrates is enriched in probiotic bacteria including bifidobacteria and lactic acid bacteria. In addition, diets that were rich in wheat bran and whole grain promoted an increase in intestinal bifidobacteria and lactobacilli (183, 184). A significant influence is held by non-digestible polysaccharides, but microbiota-accessible carbohydrates (MAC), which are not present in adequate amounts in the Western diet, which generally is based on heavily processed foods, rich in sugar, protein, fat, and different additives, and very low in fibers and micronutrients. For example, individuals on the Western diet only consume half of the recommended daily intake of dietary fiber. Recent reports demonstrate that the prevalence of these non-communicable diseases has increased dramatically in Western lifestyle countries, some of them doubling (e.g., asthma and MS) or even tripling (Crohn’s disease) in different Western European countries (155). In a multigenerational study performed on mice, the consumption of a Western-style diet aggravated the loss of microbiota diversity with a predicted loss in diversity of glycoside hydrolases, compared with a diet that was rich in MACs (155). A large study performed on a cohort of 168,999 women and 219,123 men showed that, in both genders, dietary fiber intake was significantly linked to a 22% decrease in mortality rates from infectious, cardiovascular, and respiratory diseases (185). Low-MAC content food, such as food desserts were associated with an increased incidence of asthma in children (155). Disinfectants and antibiotics generally induce a long-term decrease in bacterial diversity and, in some individuals, can affect certain particular taxa, which do not recover even months after treatment. The affected microbiota will have a decreased colonization resistance, hence allowing foreign microbes to cause permanent changes in the microbiome and varying states of disease. The outgrowth of opportunistic pathogens (pathobionts) may favor their translocation from mucosa to the extraintestinal compartment where they can initiate infectious processes (186). The majority of the antibiotics available at this moment have a broad spectrum of activity and they act not only on pathogens but also on beneficial members of the gut microbiota (187). It was reported that overexposure to antibiotics may promote the development of antibiotic resistance genes (ARGs)—studied by genomic and metagenomic approaches in the commensal microbiota and to their potential transfer to pathogenic species; they enrich phage-encoded genes and enhance the ARGs exchange between phages and bacteria (188). The repeated use of antibiotics in humans augments the reservoir of ARGs in the host microbiome, as revealed by the increased incidence of multidrug resistant (MDR) *E. coli* or vancomycin resistant enterococci (VRE) in children receiving antibiotics for respiratory infections (11). At the same time, it was revealed that limited exposure to antibiotics select the resistant pathogens (189). Recently it was shown that antibiotics change the functions of the normal gut microbiota after destroying its structure (190) by altering the diversity of microbial associated molecular patterns. After antibiotic treatment, the gut microbiota manifests resiliency and is able to present a similar composition after a long period (191). ARGs can be horizontally transferred through transformation, conjugation, and phage transduction, the mobilization promoted by mobile genetic elements such insertion sequences, integrons, and transposons (192). Several researchers have demonstrated that ARGs are present not only the microbiota of adults also in that of children and infants (193). Several antibiotic classes lead to different patterns of microbiota alterations because of their different spectrum of activity. For example, Gibson et al. demonstrated that ticarcillin-clavulanate, meropenem, and cefotaxime treatments were correlated with decreased microbiota species richness in children (194). It was also demonstrated that β-lactam combinations of beta-lactamase inhibitors and penicillins or cephalosporins determined an increase in *Proteobacteria* (specially *Enterobacteriaceae*) and *Bacteroidetes* and a decrease in *Firmicutes* as well as to a reduced microbial richness (195). By contrast, it was demonstrated that penicillin V and amoxicillin did not correlate to significant microbiota changes (196). Broad-spectrum antibiotics, like clindamycin, which are active against anaerobic species (197) were demonstrated to decrease the abundance of lactobacilli and bifidobacteria (198); macrolides have been shown to increase *Bacteroides* spp. and *Proteobacteria* levels (196) and decrease the abundance of *Actinobacteria* and *Firmicutes* taxa. Ciprofloxacin has been shown to diminish the Gram-negative facultative anaerobes, increase the abundance of the Gram-positive aerobes, and reduce the microbiota diversity; levofloxacin decreased the number of Gram-positive anaerobic microbes, including *Bifidobacteria* (197). Jakobsson et al. reported that clarithromycin decreased the number of *Actinobacteria* intrinsically resistant to metronidazole (199). Nitrofurantoin (active against Gram-negative and Gram-positive species) lead to a temporary increase in the number of *Bifidobacteria* in the gut microbiota of patients treated for uncomplicated urinary tract infections (200). Along with the multiple benefits of the uses of antibiotics in public health, agriculture, and medicine, they induce dysbiosis with negative effects on health which can remain for long periods of time after cessation of treatment. Antibiotic treatment increases the opportunities for horizontal gene transfer with crucial implications for the emergence of resistance.

## Role of Probiotics

Lactic acid-producing bacteria including *Enterococcus* spp., *Lactobacillus* spp., *Bifidobacterium* spp., but also several *Bacillus* spp., *E. coli*, and *Streptococcus* spp. strains are considered the most beneficial microbial species isolated from the human microbiota and are proposed as probiotics. Probiotics can be used to correct the antibiotics-induced disbiosis specifically in critically ill patients. The intrinsic resistance of different probiotic strains to current antimicrobials facilitates their concomitant use with specific antibacterial treatments. For example, *Lactobacillus rhamnosus* GG is constitutively resistant to metronidazole and vancomycin and is routinely used for the treatment of pseudomembranous colitis and antibiotic-associated diarrhea, while *Lactobacillus fermentum* ME-3 (DSM14241) can be used in association with ofloxacin for the treatment of *Salmonella enterica* serovar *typhimurium* infections (201, 202). Manges et al. employed a comparative metagenomic approach and reported that the development of CDI in humans is linked to an increase in *Firmicutes* and a depletion of *Bacteroidetes* phylum. It was shown that human probiotic infusion corrects the dysbiosis in CDI by replacing the depleted bacterial species and re-establishing colonization resistance (203, 204). Also, in their study, Khodaii et al. evaluated the effects of cell-free supernatants of cultures belonging to 16 strains of lactobacilli and bifidobacteria on the invasive capability of enteroinvasive *Escherichia coli* (EIEC) strain. Thus, the treatment of the pathogen with cell-free supernatants prevented the EIEC strains invasion of CaCo-2 and T84 cells. They suggested that probiotics prevent invasion of EIEC into the small and large intestine not by competing with adhesins receptors, but by producing some metabolites that changes the environment, cell barrier, or gene expression (205). Lactic acid bacteria produce bacteriocins in a cell density-dependent manner and utilize a molecular QS regulatory mechanism. Bacteriocin production is an inducible mechanism and requires the extracellular accumulation of certain chemical messengers (AI1 and AI2) (206). So, for specific purposes either probiotic products (live cells/dead) or probiotic soluble molecules (antimicrobials, QSI molecules) can be used. Not only microorganisms are producing QS molecules and QSIs, but also plants are able to interfere with bacterial communication and processes controlled by QS mechanism, as an expression of their antiinfectious defense mechanisms and these inhibitory molecules with demonstrated *in vitro* activity and can be used alone or in combinations as an alternative/complementary antiinfectious strategy (60). Probiotics confer health benefits through the modulation of pro- and anti-inflammatory responses. Studies reported that cell surface molecules of *Lactobacillus* strains exhibit TNF-α-inducing activities in macrophages *via* TLR2 signaling (179). *In vitro* and *in vivo* studies suggest that *Propionibacterium* species can be used as probiotics, with many potential health benefits, modulating gut microbiota composition and gut activities (207, 208). Dairy propionic bacteria can impact the gut microbiota by favoring the growth of symbiotic bacteria such as *Bifidobacteria*, or by inhibiting the *in vitro* adherence of some pathogens such as *H. pylori* (209), *Salmonella enterica* serovar Enteritidis and enteropathogenic *Esherichia coli* strains to different cell lines (210, 211). Also, clinical studies reported the beneficial outcome of combining dairy propionibacteria with other probiotic bacteria in order to modulate the host immune system (212). Thus, two probiotic bacteria, *Propionibacterium freudenreichii* spp. *shermanii* (PJS) and *Lactobacillus rhamnosus* GG (GG) were tested for immunomodulatory response in the mouse intestine, using fat-fed ApoE*3Leiden mice. It was shown that mice receiving PJS and GG harbored significantly lower intestinal mast cells compared to the control. Also, GG increased intestinal IL-10, whereas PJS lowered the intestinal immunoreactivity of TNF-α. Probiotics represent also a very efficient preventive therapy against necrotizing enterocolitis (NEC), the potential mechanisms of this effect being experimentally demonstrated. Thus, oral administration of *Bifidobacterium bifidum* cells decreased the levels of ileal claudin-3 and occludin in neonatal rats with NEC (213). Also, bacteria-free conditioned media harvested from probiotics such as *Bifidobacterium infantis, Lactobacillus plantarum*, and *Lactobacillus acidophilus* administered in single or in multiple combinations, may confer protection against NEC, by their anti-inflammatory and cytoprotective properties, and by improving intestinal barrier function (214, 215). A new approach in the probiotic therapy is represented by the bioengineered probiotics. Probiotic strains can be employed as vehicles for expressing foreign genes. In line with this, Culligan et al. analyzed the advantages of recombinant probiotics in treating enteric infection (216) but such probiotic strains are regarded as genetically modified organisms, raising ethical issues related to their use (217).

## Conclusion and Perspectives

The application of macroecological concepts to the gut microbiota indicates that the microbiota biodiversity can serve as an important measure of eubiosis status.

The easy access and possibility to modulate the microbiota makes it a good target for both establishing a link between certain patterns of gut microbiota and the physiological/pathological status. Due to the complexity and inter-individual differences of human microbiota, the identification of microbial colonization profiles specifically associated with certain disorders, as well as the characterization of microbial metabolic pathways related to health and disease state still remains a challenge.

The human microbiota interacts at multiple levels with the immune system and the alteration of this crosstalk could be involved in the pathophysiological mechanisms of the host and can further be exploited to develop clinical therapies for some immunological disorders, such as inflammatory and autoimmune diseases, allergies, cancer, dysbiosis, and opportunistic infections. This could lead to the development of potential biomarkers allowing to implement personalized healthcare strategies and to identify new tools for prevention, screening, and treatment.

However, the very diverse actors of these complex interactions (bacterial species, microbial products, host receptors, signaling molecules, and molecular pathways) as well as diet influence are still to be uncovered. Related to diet, it has long been seen as an adjuvant of medication, but recent research data are offering arguments for the use of food to efficiently modulate the microbiota and to develop microbiota-based interventional therapies or personalized diets, tailored in accordance with the host genetic background, microbiome, metabolome, as well as nutrient intake and habitual food consumption.

In perspective, we can target a homeostatic status of microbiota and host health, by designing microbiota-based therapeutics, but also by reducing antimicrobials consumption and assuring a diverse and balanced diet for the health of both host metabolism and its microbiota. In order to achieve this desideratum, an educational component, assuring the proper understanding of microbiota structure and roles in host health and disease, is absolutely needed to convince people about the necessity to introduce some long-term changes in their lifestyle, instead of turning to short time therapeutic interventions in emergency situations.

## Author Contributions

VL and GP drafted the manuscript. L-MD, GP, CC, IG, AH, AP and LP wrote the manuscript. GP made the figures; MC wrote and corrected the manuscript.

## Conflict of Interest Statement

The authors declare that the research was conducted in the absence of any commercial or financial relationships that could be construed as a potential conflict of interest.
